# Cardiac Sarcoidosis: A Unique Presentation

**DOI:** 10.7759/cureus.27295

**Published:** 2022-07-26

**Authors:** Mihir Odak, Kameron Tavakolian, Steven Douedi, Ndausung Udongwo, Islam Elkherpitawy, Hani Douedi, Natasha Campbell

**Affiliations:** 1 Internal Medicine, Jersey Shore University Medical Center, Neptune City, USA; 2 Cardiology, Community Medical Center, Toms River, USA

**Keywords:** cardiovascular magnetic resonance imaging (cmr), infiltrative disease, arrhythmia, cardiac sarcoidosis, sarcoidosis

## Abstract

Isolated cardiac sarcoidosis is a rare subset of sarcoidosis, a systemic autoimmune condition primarily found in African American females. The manifestations of cardiac sarcoidosis include atrioventricular and bundle branch blocks, arrhythmias, heart failure, and pericardial effusions, although these complications occur at varying prevalence. The diagnosis of cardiac sarcoidosis requires several different criteria; however, recent literature has focused heavily on imaging modalities such as cardiac magnetic resonance imaging. We present a case of a 42-year-old Caucasian male who was found to have unexplained cardiac arrhythmias and ultimately diagnosed with cardiac sarcoidosis by imaging modalities.

## Introduction

Sarcoidosis is an uncommon autoimmune disorder primarily found in African American females [1,2]. Of patients diagnosed with this condition, cardiac sarcoidosis (CS) is found in around 7% of affected individuals, and isolated cardiac sarcoidosis (ICS) is even less likely, accounting for around 27-54% of all diagnosed CS cases [1]. Cardiac arrhythmias such as bradycardia, ventricular arrhythmias, and even sudden cardiac death are common presentations of this debilitating disease [3]. We present a case of a 42-year-old Caucasian male who presented to the emergency department (ED) due to intermittent episodes of chest pain and shortness of breath that started two months prior to admission. Cardiac magnetic resonance imaging (CMR) revealed findings consistent with CS. To the best of our knowledge, there are only a few reported cases of ICS in quadragenarian Caucasian males.

## Case presentation

A 42-year-old Caucasian male with a medical history of vertigo, orthostatic syncope, anxiety, gastroesophageal reflux disease, and chronic back pain presented to the ED with complaints of chest pain that started two months prior to admission. The patient also reported testing positive for severe acute respiratory syndrome (SARS-CoV-2) around the time his symptoms started. The pain was 2/10 in severity, intermittent (lasting 30 minutes), dull in quality, with no aggravating or alleviating factors, and with radiation to the left jaw. His symptoms worsened over the past five to six days and were associated with bilateral tingling/numbness in the upper extremities, non-localized headache, cough, shortness of breath, night sweats, palpitations, dizziness, and wheezing. Of note, he was admitted for similar symptoms two weeks ago. Imaging tests including computed tomography angiography (CTA) of the chest, CT of the spine, and CT of the head were unremarkable. Cardiology and gastroenterology workups were unremarkable before discharge. He denied any family history of heart disease. He smoked one pack of tobacco per day for around 18 years, as well as marijuana occasionally.

**Figure 1:**
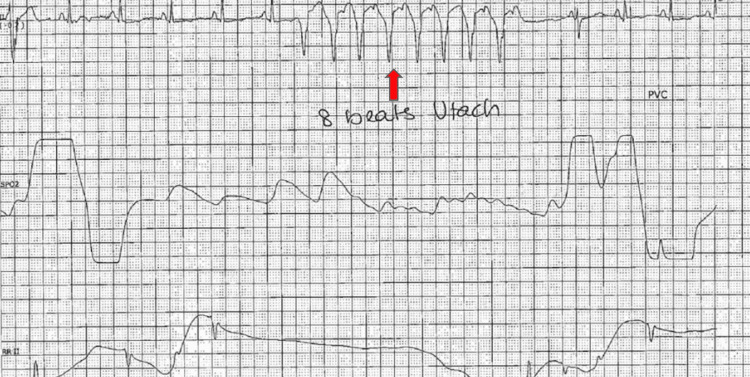
Telemetry monitor. Eight beats of nonsustained ventricular tachycardia (red arrow) were appreciated on the telemetry monitor.

**Figure 2:**
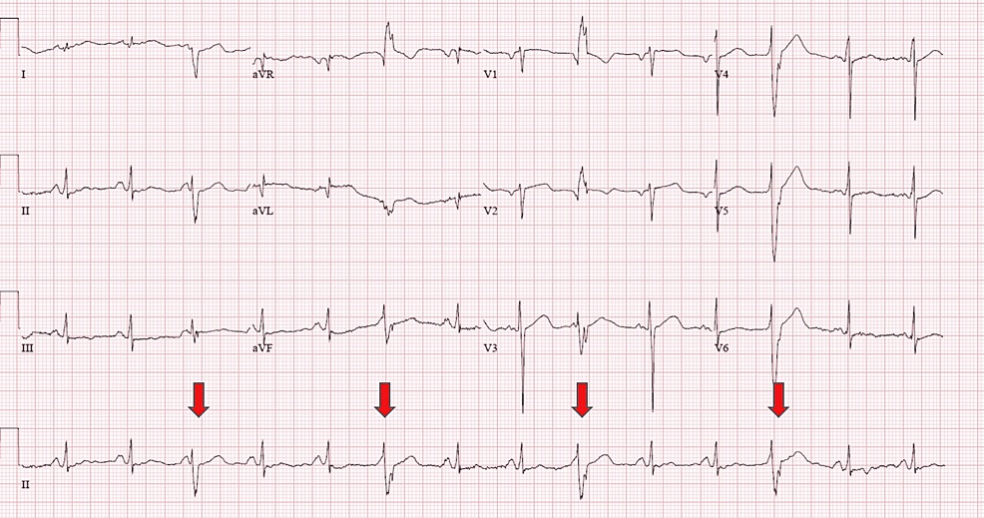
Electrocardiogram. Normal sinus rhythm, heart rate of 85 beats per minute, premature ventricular complexes, and trigeminy (red arrows), with no ST or T wave changes were noted.

On presentation, his initial vitals were as follows: blood pressure of 132/74 mmHg, heart rate of 85 beats per minute, respiratory rate of 16 breaths per minute, temperature of 98.3℉, and oxygen saturation of 99% on ambient air. On physical examination, he was not in any acute distress, and his cardiovascular examination was unremarkable. On auscultation of the lungs, wheezing was noted in the left upper and right middle lung fields. While in the ED, 8 beats of nonsustained ventricular tachycardia were appreciated on the telemetry monitor (Figure 1). Electrocardiogram (EKG) revealed normal sinus rhythm, heart rate of 85 beats per minute, premature ventricular complexes, and trigeminy, with no ST or T wave changes (Figure 2). The patient’s presenting laboratory data are given in Table 1.

**Table 1:** Laboratory data on admission

Serum	Results	Reference range
White blood cells (x10^3^/uL)	8.8	4.5–11.0
Hemoglobin (g/dL)	12.3	12.0–16.0
Mean corpuscular volume (fL)	91.4	80.0–100.0
Platelet count (x10^3^/uL)	291	140–450
Glucose (mg/dL)	101	136–145
Blood urea nitrogen (mg/dL)	7	5–25
Creatinine (mg/dL)	0.85	0.44–1.0
Sodium (mmol/L)	139	135–146
Potassium (mmol/L)	3.5	3.5–5.2
Chloride (mmol/L)	104	96–110
Calcium (mg/dL)	9.2	8.5–10.5
Magnesium (mg/dL)	1.9	1.3–2.5
Bicarbonate (mmol/L)	27	24–31
Alkaline phosphatase (U/L)	84	38–126
Total protein (g/L)	6.6	6.0–8.0
Albumin (g/dL)	3.7	3.5–5
Bilirubin, total (mg/dL)	0.9	0.2–1.3
Aspartate aminotransferase (U/L)	32	10–42
Alanine aminotransferase (U/L)	66	10–60
Troponin (ng/mL)	0.03	<0.04

**Figure 3:**
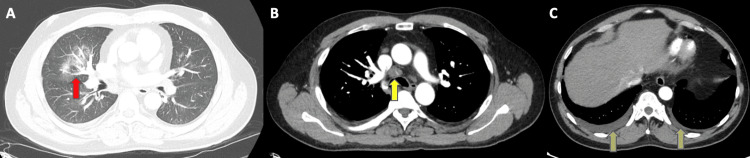
Computed tomography angiography. Computed tomography angiography of the chest demonstrating patchy areas of consolidation (red arrow), extensive mediastinal (pretracheal) lymphadenopathy (yellow arrow), and bilateral pleural effusions (green arrows).

CTA of the chest revealed patchy areas of consolidation, extensive mediastinal lymphadenopathy, and bilateral pleural effusions (Figure 3). Thrombolysis in myocardial infarction (TIMI) score was 3. He was admitted and treated for non-ST elevation myocardial infarction with 4,000 units of IV heparin, aspirin 324 mg, carvedilol 3.125 mg twice a day, azithromycin 500 mg (IV), and ceftriaxone 1 g (IV).

**Figure 4:**
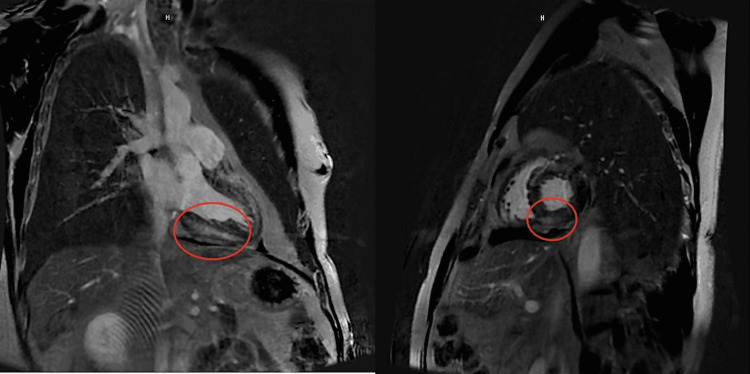
Cardiac magnetic resonance imaging. Cardiac magnetic resonance imaging with and without contrast showing a mildly reduced systolic function of 47.7%, and an extensive mid-wall myocardial fibrosis in the anterior, septal/apical, inferior, and inferoseptal walls (red circles) on late gadolinium enhancement

Results from echocardiography revealed an ejection fraction of 55-60%, mild tricuspid/mitral regurgitation, a small circumferential pericardial effusion, and no regional wall abnormalities. Left heart catheterization was performed, which revealed an absence of coronary artery disease with elevated left ventricular end-diastolic pressure. CMR with/without contrast revealed a mildly reduced systolic function of 47.7%, and an extensive mid-wall myocardial fibrosis in the anterior, septal/apical, inferior, and inferoseptal walls on late gadolinium enhancement (Figure 4). These findings were consistent with inactive CS. He was discharged in a stable condition with a plan for outpatient cardiology and rheumatology follow-up, as well as a positron emission tomography (PET) scan.

## Discussion

Sarcoidosis is a multisystem inflammatory disorder that can manifest in cardiovascular tissue as CS [4]. It is caused by granulomatous inflammation, which then ultimately results in dysfunction depending on the specific area of the heart that is affected, with edema and fibrosis being the causative mechanisms. While any area of the heart can be infiltrated by these granulomas, the left ventricle (LV) is most commonly involved [5]. The resulting inflammatory changes to tissue, fibroblast and collagen accumulation, and scarring lead to changes in cardiac physiology including impaired contractility and atrioventricular block. The Case Controlled Etiologic Sarcoidosis Study (ACCESS) has also suggested a genetic predisposition to developing sarcoidosis, with a relative risk of developing the condition of 5 as compared to the control group. Sarcoidosis has an estimated prevalence of up to 0.04% in the United States and European countries, with a particular predominance in African American individuals. Although sarcoidosis is rare by these measures, CS typically affects up to 7% of patients with sarcoidosis, suggesting an extremely uncommon syndrome [3,6]. The manifestations of CS include atrioventricular and bundle branch blocks, arrhythmias, heart failure, and pericardial effusions, although these manifestations occur at varying prevalence [7].

Several diagnostic criteria exist to suggest CS; one such criterion is the Japanese Ministry of Health and Welfare criteria, which suggests CS in the presence of findings suggestive of pulmonary or ophthalmologic sarcoidosis and at least two major criteria, including depressed ejection fraction and late gadolinium enhancement (LGE) on CMR, such as in our patient [8]. The diagnosis of CS, therefore, relies heavily on imaging modalities. CMR has become a mainstay of visualization of infiltrative and inflammatory processes in the heart [9,10]. One particular benefit of CMR is its ability to detect layers of involvement of cardiac tissue in new-onset cardiomyopathy. Late-gadolinium enhancement has been suggested to have varying findings on CMR in ischemic heart disease and nonischemic cardiomyopathy [10]. While ischemic disease results in LGE in transmural and subendocardial patterns, CS generally results in subepicardial and intramural LGE patterns, which can aid in differentiation [10]. Recent literature has also suggested the role of CMR over PET imaging, given the increased spatial resolution offered by CMR compared to PET, which can aid in identifying smaller lesions. Although clinical manifestations of CS typically occur with larger lesions, it remains to be seen how early identification of microscopic granulomatous lesions impacts the mortality rate of CS [11].

## Conclusions

CS, particularly ICS, is a debilitating condition; it has limitations in management options aside from steroid therapy. Recent literature has suggested the role of CMR and has questioned the role of PET imaging in the diagnosis of ICS. Our hope in presenting this case is to emphasize the role of CMR imaging in new-onset cardiomyopathy and to encourage a high degree of suspicion for CS, despite its rarity in reports.
